# Amphotericin B and Curcumin Co-Loaded Porous Microparticles as a Sustained Release System against *Candida albicans*

**DOI:** 10.3390/molecules27103079

**Published:** 2022-05-11

**Authors:** Baiji Xue, Yanhua Yu, Guoqiang Peng, Mengmeng Sun, Peng Lv, Xuefeng Li

**Affiliations:** 1 School of Basic Medical Sciences, Baicheng Medical College, Baicheng 137000, China; xbj@bcmc.edu.cn (B.X.); yyh@bcmc.edu.cn (Y.Y.); pgq@bcmc.edu.cn (G.P.); smm@bcmc.edu.cn (M.S.); 2 The Sixth Affiliated Hospital of Guangzhou Medical University (Qingyuan People’s Hospital), Sino-French Hoffmann Institute, School of Basic Medical Sciences, Guangzhou Medical University, Guangzhou 511436, China; 3 Shenzhen Luohu People’s Hospital, The Third Affiliated Hospital of Shenzhen University, Shenzhen 518001, China

**Keywords:** amphotericin B, curcumin, PLGA MPs, antifungal activity, biofilms

## Abstract

Amphotericin B (AMB) is an antifungal drug used for serious fungal infections. However, AMB has adverse reactions such as nephrotoxicity, which limit the clinical application of AMB alone or in combination with other antifungal drugs. Nano or micro drug delivery systems (DDS) have been proven to be effective in reducing the toxic and side effects of drugs. Further, the combination of AMB with other compounds with antifungal activity, such as curcumin (CM), may enhance the synergistic effects. Herein, AMB and CM were co-loaded into porous poly (lactic-co-glycolic acid) (PLGA) microparticles (MPs) to prepare AMB/CM-PLGA MPs. The AMB/CM-PLGA MPs showed a remarkably reduced hemolysis (62.2 ± 0.6%) compared to AMB (80.9 ± 1.1%). The nephrotoxicity of AMB/CM-PLGA MPs is significantly lower than that of AMB. In vitro, AMB/CM-PLGA MPs had better inhibitory effects on the adhesion and biofilm formation of *Candida albicans* compared with AMB. Experiments on mice infected with *C. albicans* showed that AMB/CM-PLGA MPs have a better therapeutic effect than AMB in vivo. In summary, AMB/CM-PLGA MPs may be a novel and promising therapeutic candidate for fungal infection.

## 1. Introduction

In recent years, the infection and mortality rate of candidiasis have significantly increased due to tumor chemoradiotherapy, the widespread use of antibiotics, and the increase in the number of immunocompromised patients [1]. *C. albicans* is the most common fungal species that causes candidiasis. It can cause cutaneous and mucosal infections such as vaginal infections, thrush, and life-threatening invasive infections [2]. Among the 20,788 isolates of invasive *Candida* collected from around the world for 20 years (1997–2016) in the SENTRY Antifungal Surveillance Program, 46.9% were *C. albicans* [3]. In Asian countries, *C. albicans* is the main pathogen causing candidemia. The clinical isolation rate of *C. albicans* was the highest (41.3%) [4]. *C. albicans* is even one of the most common coinfection fungi in COVID-19 patients [5]. *C. albicans* is also a dimorphic fungus, with two forms of yeast and filamentous hyphae. Biofilm is one of the core virulence factors that help to enhance its pathogenicity and bring great trouble to clinical treatment [6].

Amphotericin B (AMB) is a polyene antibiotic naturally produced by *actinomycete Streptomyces nodosus*. Due to its broad-spectrum antifungal effect and low incidence of clinical resistances, it is of a particular importance in clinical practice for treating fungal diseases, including candidiasis. Its mechanism of action is that AMB interacts with the ergosterol of susceptible fungal cell membranes and alters its structure and permeability [7]. However, the application of AMB is limited because it is severely toxic to normal tissues and can cause various side effects, including infusion-related toxicity and chronic toxicity. In order to overcome the shortcomings of AMB, several drug delivery systems (DDS) have been developed, such as lipid formulations of AMB and AMB solubilized with sodium deoxycholate [8,9]. Furthermore, porous microparticles (MPs) were considered to be promising. MPs have the characteristics of large geometric diameter and low bulk density, which can reduce the phagocytosis of alveoli and the tendency of particle aggregation, thereby improving the efficiency of atomization. MPs based on biocompatible and biodegradable polylactide-glycolic acid (PLGA) have been suggested as potential sustained-release carriers for pulmonary drug delivery [10,11].

Curcumin (CM) is a well-known dietary pigment derived from turmeric; it has various pharmacological activities. Importantly, clinical trials have shown that CM is safe in humans and that oral CM 12 g/d has minimal toxicity. Recently, many studies on CM have confirmed that CM inhibits the growth of fungi. In addition, studies have shown that CM can synergize with conventional antifungal agents [12]. However, the drug has poor water solubility and low bioavailability, so it is necessary to improve the dosage form. It has been reported that DDS can be loaded with CM to increase water solubility [13]. Herein, we intend to utilize PLGA to co-load AMB and CM to prepare porous microparticle AMB/CM-PLGA MPs. AMB toxicity in AMB/CM-PLGA MPs was assessed by hemolysis assay and nephrotoxicity assay. In addition, the anti-*C. albicans* activities of AMB/CM-PLGA MPs and AMB were comparatively analyzed in vivo and in vitro. We expect to obtain an AMB-loaded DDS that improves the anti-*C. albicans* activity of AMB while reducing its toxicity, which may be a novel and promising drug candidate for the treatment of fungal infections.

## 2. Results and Discussion

### 2.1. Microparticles Characteristics, Drug Loading Content (DLC), and Drug Loading Efficiency (DLE)

**Figure 1:**
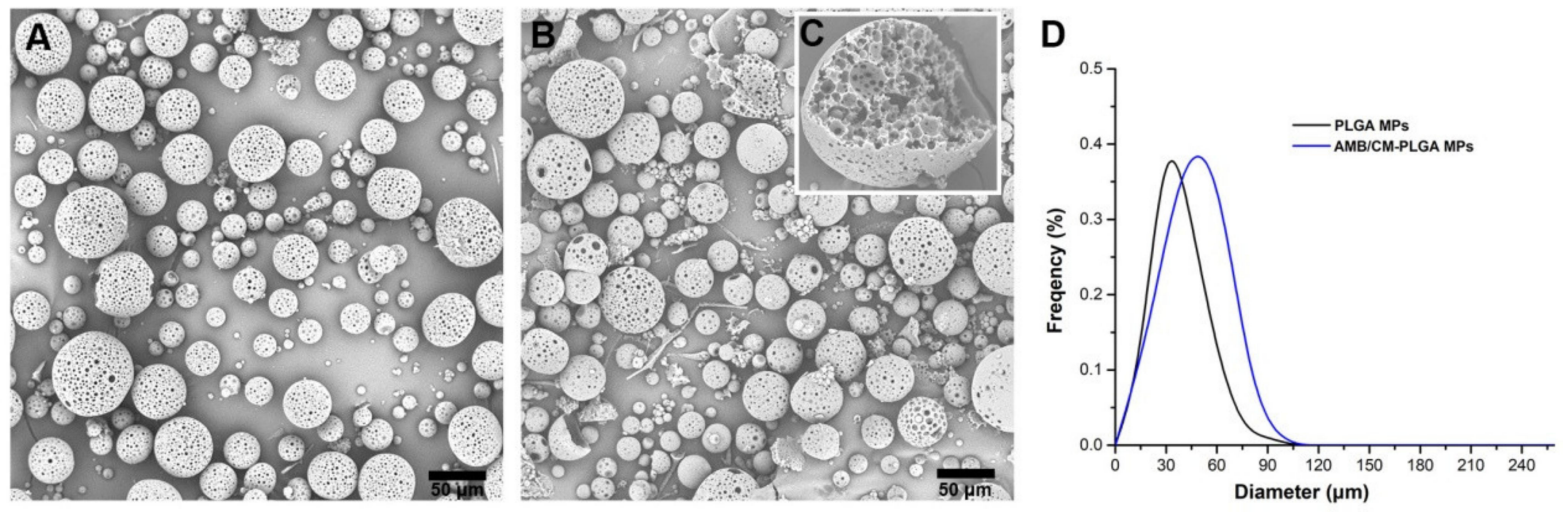
Morphology of the porous particles observed by SEM: (**A**) PLGA MPs; (**B**) AMB/CM-PLGA MPs; (**C**) cross-section of AMB/CM-PLGA MPs. Particle size distributions of the porous particles: (**D**) PLGA MPs and AMB/CM-PLGA MPs.

Porous PLGA MPs and AMB/CM-PLGA MPs were prepared by double emulsion solvent evaporation technology. The morphologies of PLGA MPs and AMB/CM-PLGA were observed by SEM. The PLGA MPs have a spherical shape and a large number of pores on the surface (Figure 1A). The morphologies of the AMB/CM-PLGA MPs had no obvious change compared to PLGA MPs after loading AMB and CM into the MPs (Figure 1B). As shown in Figure 1C, the cross-sectional area of AMB/CM-PLGA MPs was full of pores. The pore size observed by calculation varies from about 110 to 500 nm. The particle size distributions are shown in Figure 1D. The particle sizes of the PLGA MPs were approximately 30.5 ± 5.5 μm, but after loading AMB and CM into PLGA, it slightly increased to approximately 47.5 ± 4.2 μm. In addition, the SPAN values of PLGA MPs and AMB/CM-PLGA MPs were 0.66 and 0.79, respectively, which indicated that the prepared microparticles had better uniformity.

The AMB and CM were loaded into PLGA via the double emulsion method. From Table 1, when the feeding ratio was 14:2:4, AMB/CM-PLGA MPs provided a high AMB loading efficiency of 60.8% and an AMB loading content of 6.08 wt%, with a CM loading efficiency of 72.5% and a CM loading content of 14.5 wt%, indicating that AMB/CM-PLGA MPs had excellent drug-loading capacity for both AMB and CM at the feeding ratio. Higher drug feeding ratios can produce slightly higher DLC, while DLE was significantly reduced. When the feeding ratio was set at 12:2:6, the CM loading efficiency was reduced to 64.0%. Next, AMB/CM-PLGA MPs with an AMB loading efficiency of 6.08 wt% and a CM loading content of 14.5 wt% were applied to further studies in order to obtain a rational DLC while maintaining a high DLE. The results obtained here are similar to our previous report [14] and also advocate that CM’s concentration might be adjusted to get the desired DLC and DLE.

### 2.2. AMB and CM Release from the AMB/CM-PLGA MPs

**Figure 2:**
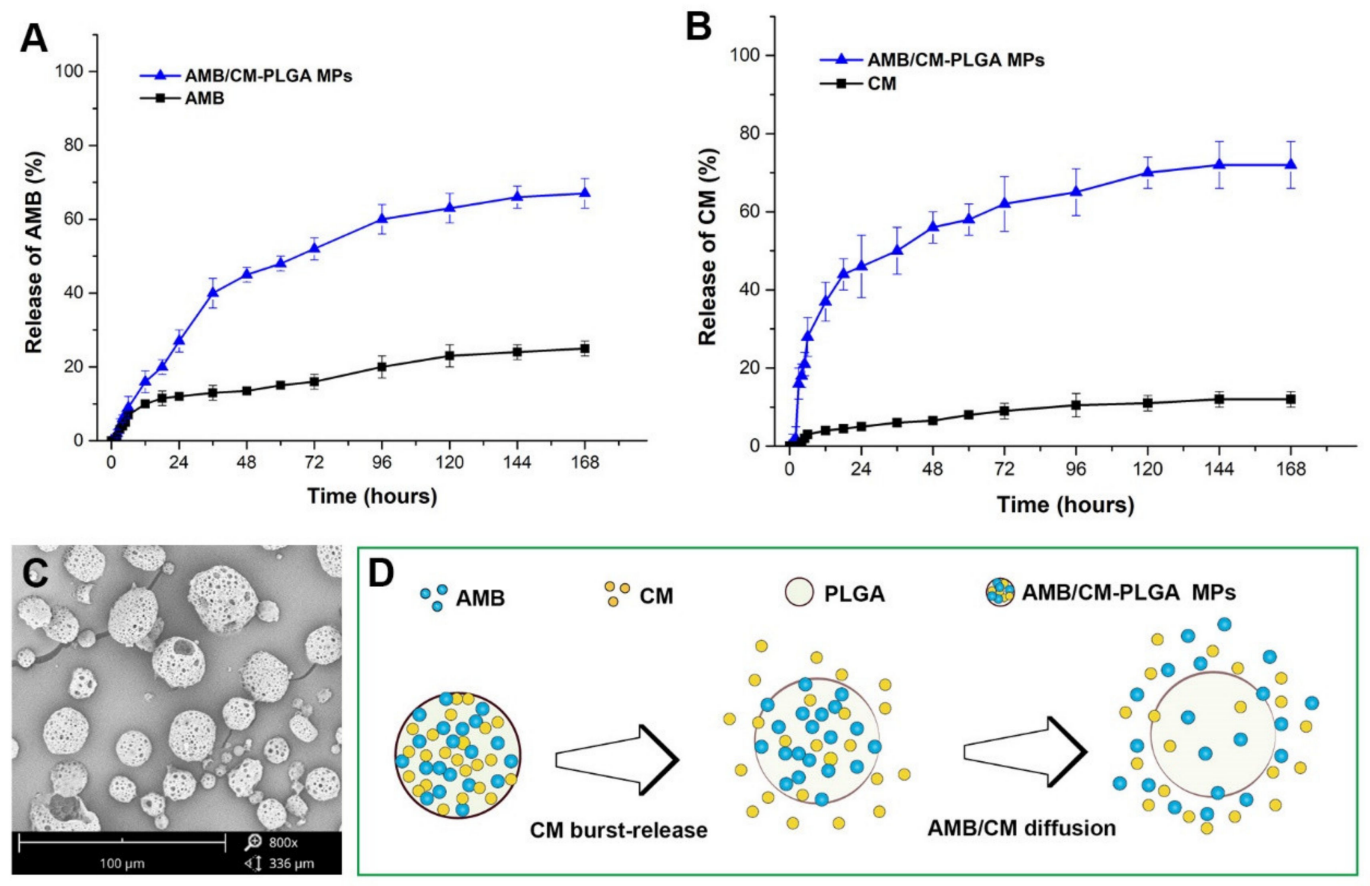
AMB and CM release from the AMB/CM-PLGA MPs. (**A**) In vitro drug release profiles of AMB within 168 h. (**B**) In vitro drug release profiles of CM within 168 h. (**C**) AMB/CM-PLGA MPs were degraded within 24 h. (**D**) Proposed mechanism of sequential drug release.

AMB and CM were released gradually from AMB/CM-PLGA MPs. The release rate of encapsulated AMB was obviously increased after loading into PLGA compared with AMB (Figure 2A). Similarly, AMB/CM-PLGA MPs can improve the release rate of encapsulated CM (Figure 2B); AMB/CM-PLGA MPs displayed higher cumulative CM release (69%) compared with CM (9%) within 168 h. As the incubation time in PBS was prolonged, AMB/CM-PLGA MPs gradually degraded, and it was found that the surface pores became larger, which may be due to the decomposition of PLGA (Figure 2C) [15,16]. Moreover, the release rate of CM was faster than encapsulated AMB within 12 h, which revealed that the incorporated drugs may be released sequentially (Figure 2D). As seen in Figure 2A,B, the drug release curve of AMB/CM-PLGA MPs conformed to the Korsmeyer–Peppas drug release model. Since the value of n was less than 0.45, the drug release mechanism of AMB/CM-PLGA MPs can be explained by Fick’s diffusion [17].

### 2.3. In Vitro Hemolytic Assay

**Figure 3:**
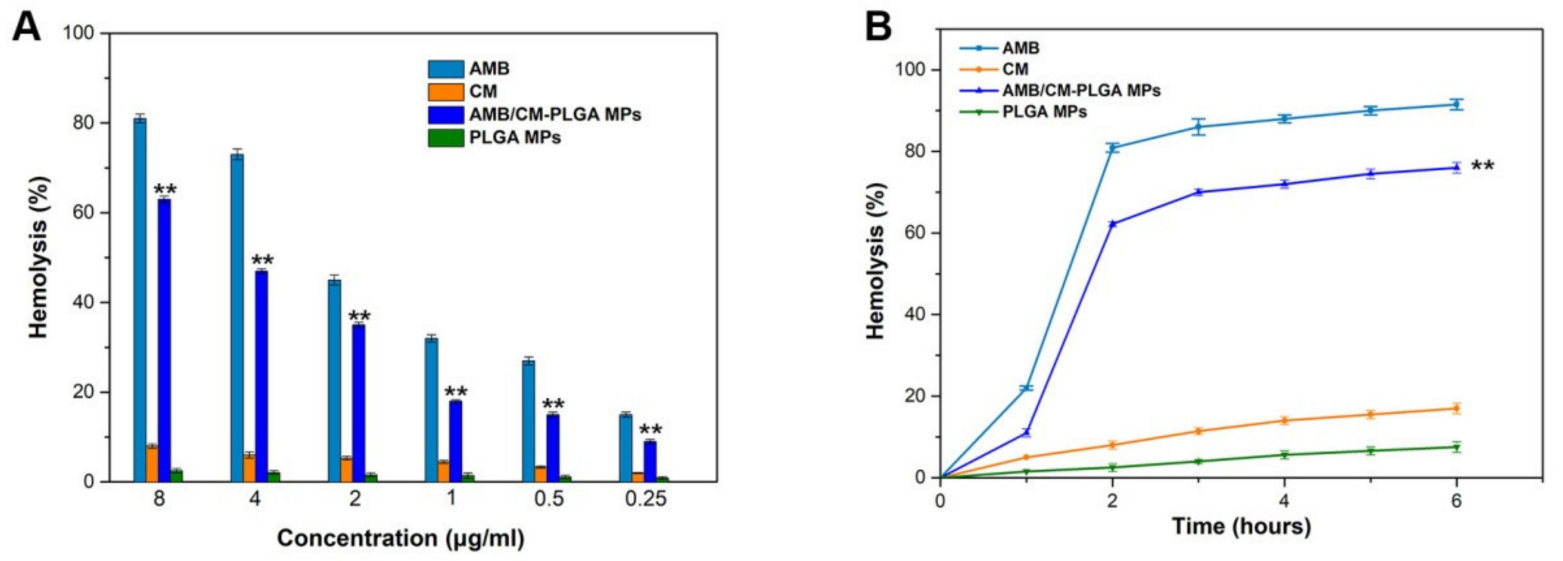
In vitro hemolytic assay. (**A**) Hemolysis of red blood cells following incubation with AMB, CM, AMB/CM-PLGA MPs, and PLGA MPs for 2 h at final AMB concentrations of 0.25 to 8 μg/mL. (**B**) Hemolysis of red blood cells following incubation with AMB and AMB/CM-PLGA MPs with the concentration of 8 μg/mL at different times. ** *p* < 0.05.

Hemolysis was a commonly used parameter to assess the toxicity of AMB [14]. After incubation for 2 h, AMB (8 μg/mL) resulted in 80.9 ± 1.1% of hemolysis (Figure 3A). Compared to AMB, the AMB/CM-PLGA MPs caused greatly reduced hemolysis (62.2 ± 0.6%) at identical AMB concentrations. At the same time, the hemolysis of CM and PLGA MPs was very low, especially PLGA MPs. From Figure 3B, it can be seen that AMB caused more severe hemolysis (21.1 ± 0.2%) compared with AMB/CM-PLGA MPs (10.1 ± 0.3%) within 1 h. AMB/CM-PLGA MPs delayed the occurrence of hemolysis and the onset of hemolysis to at least 6 h at a low hemolysis degree (14.1 ± 0.5%) (no more than 15%). These indicated that the hemolytic activity of AMB/CM-PLGA MP was lower than that of AMB because blood stability was improved [18].

### 2.4. In Vivo Nephrotoxicity

**Figure 4:**
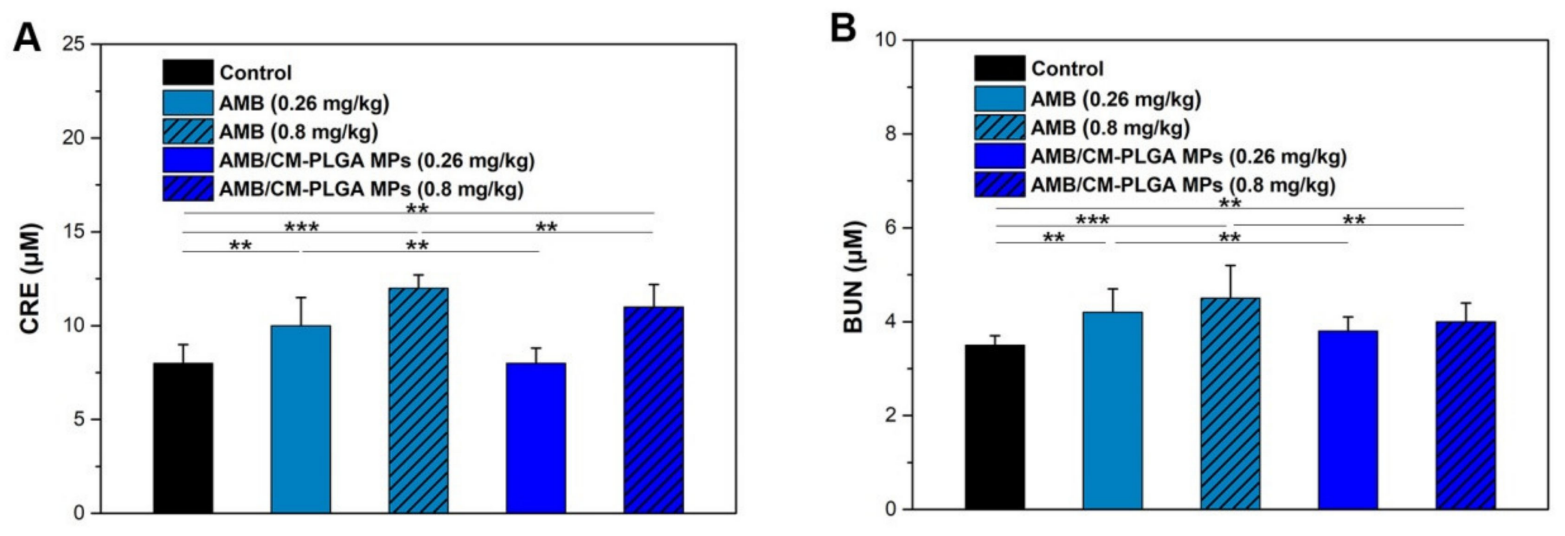
In vivo nephrotoxicity assay. CRE (**A**) and BUN (**B**) in plasma after treatment of AMB (0.26 or 0.8 mg/kg) and AMB/CM-PLGA MPs (0.26 or 0.8 mg/kg), respectively. ** *p* < 0.05. *** *p* < 0.01.

The levels of blood creatinine (CRE) and urea nitrogen (BUN) are important parameters for investigating nephrotoxicity [19,20]. Herein, the nephrotoxicity of AMB and AMB/CM-PLGA MPs was studied by measuring levels of CRE and BUN. CRE and BUN levels were measured by using a biochemical analyzer after treatment with AMB or AMB/CM-PLGA MPs. The treatment of AMB (0.26 mg/kg) significantly increased the CRE level compared with the control group. However, the CRE level in the AMB/CM-PLGA MPs group (0.26 mg/kg, which was the AMB concentration) had no obvious changes compared with that of the control group (Figure 4A). Moreover, the treatment of AMB/CM-PLGA MPs (0.8 mg/kg) significantly decreased the CRE content compared with the AMB group (0.8 mg/kg). In addition, the BUN level of the AMB group was higher than that of the AMB/CM-PLGA MPs group at the same dose of AMB (Figure 4B). These results indicate that the nephrotoxicity of AMB/CM-PLGA MPs was lower than that of AMB, which was similar to our previous report [14]. The hemolysis and nephrotoxicity assays proved that we had obtained a drug loading platform, AMB/CM-PLGA MPs, to reduce the toxicity of AMB. Next, we will explore whether the antifungal activity of the AMB/CM-PLGA MPs is also improved.

### 2.5. Analysis of the Antifungal Activity In Vitro

The antifungal activities of AMB, CM, and AMB/CM-PLGA MPs against *C. albicans* were detected following the CLSI M38-A method in vitro. The MIC was defined as the lowest reagent concentration that inhibited the growth of the tested strain. From Table 2, it can be seen that as an effective drug for the treatment of candidiasis, AMB exhibited high activity against tested strains at 0.5 μg/mL. CM also had antifungal activity against *C. albicans* ATCC 90028 and *C. albicans* ATCC 10231, with MICs of 128 and 256 μg/mL, respectively. This result is consistent with our previous report, which described the effect of CM on *C. albicans* [13]. Table 2 also shows that AMB/CM-PLGA MPs display anti-*C. albicans* activity against tested strains at 8 μg/mL. The DLC of AMB in AMB/CM-PLGA MPs was 6.08%, the value (8 μg/mL) actually contained AMB at a concentration of 0.4864 μg/mL, which was slightly lower than 0.5 μg/mL. Since the two values of 0.4864 μg/mL and 0.5 μg/mL were so close, it was not clear from the MIC experimental results that AMB/CM-PLGA MPs exhibited better anti-*C. albicans* activity than AMB alone.

**Figure 5:**
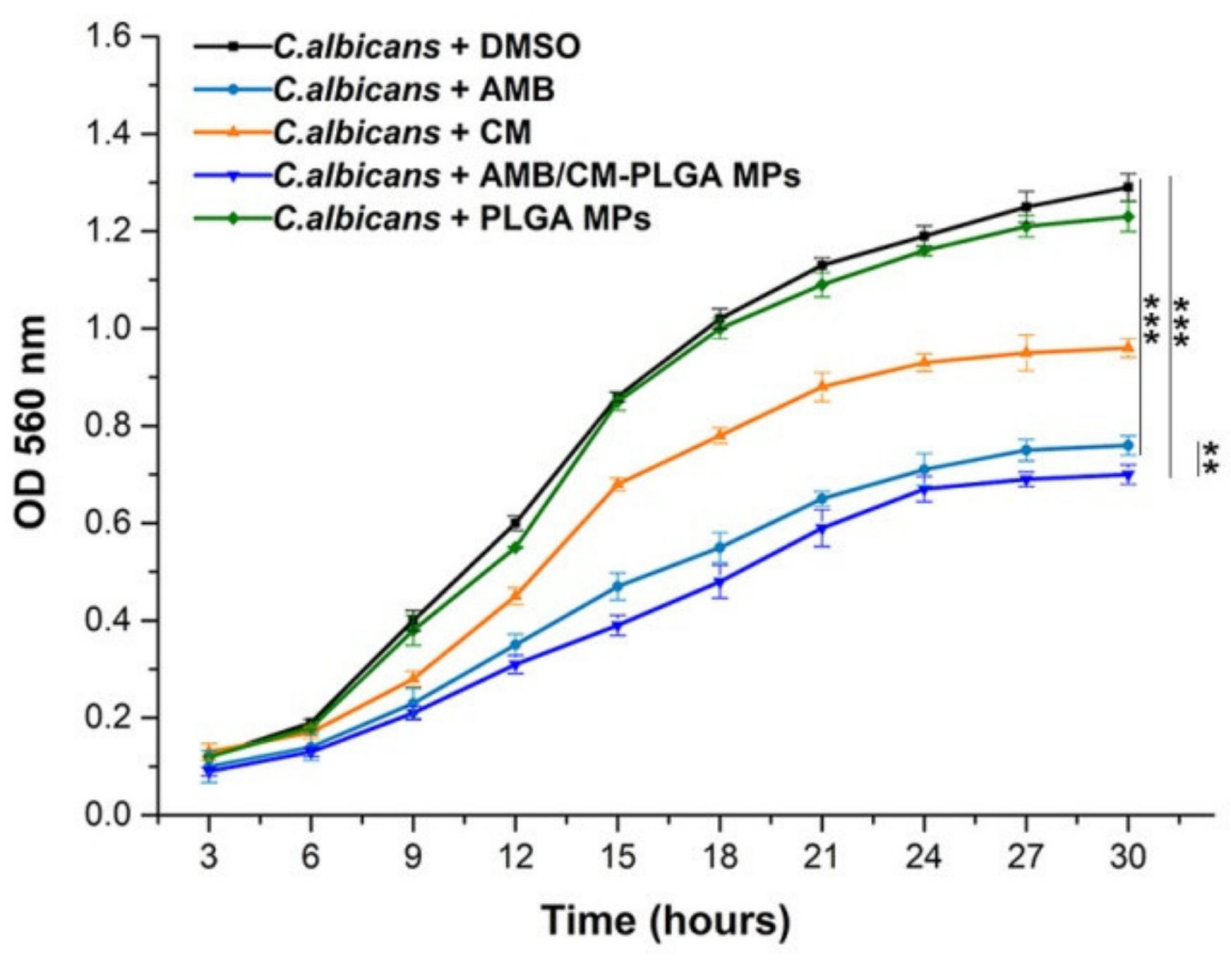
Growth kinetics of *C. albicans* in the presence of antifungal agents. Effect of AMB, CM, and AMB/CM-PLGA MPs at their MIC/2 concentrations on the growth of *C. albicans*. Growth curve plotted against absorbance at 560 nm. ** *p* < 0.05. *** *p* < 0.01.

The growth curves of *C. albicans* in the presence of AMB, CM, and AMB/CM-PLGA MPs at MIC/2 concentrations are shown in Figure 5. It shows that the exponential phases of *C. albicans* were significantly suppressed and delayed by AMB, CM, and AMB/CM-PLGA MPs, especially AMB and AMB/CM-PLGA MPs. Furthermore, the average decrease in optical density of AMB/CM-PLGA MPs was more significant than AMB at 30 h.

### 2.6. Adhesion Assay and Biofilm Assay

Adhesion between microorganisms and host cells is the premise of colonization and infection. Biofilm is one of the key factors for infection and drug resistance [21,22]. Herein, through mouse lung epithelial cell (PEC) adhesion assay and biofilm assay, the inhibitory effects of AMB, CM, AMB/CM-PLGA MPs, and PLGA MPs on *C. albicans* ATCC 90028 are further explored.

**Figure 6:**
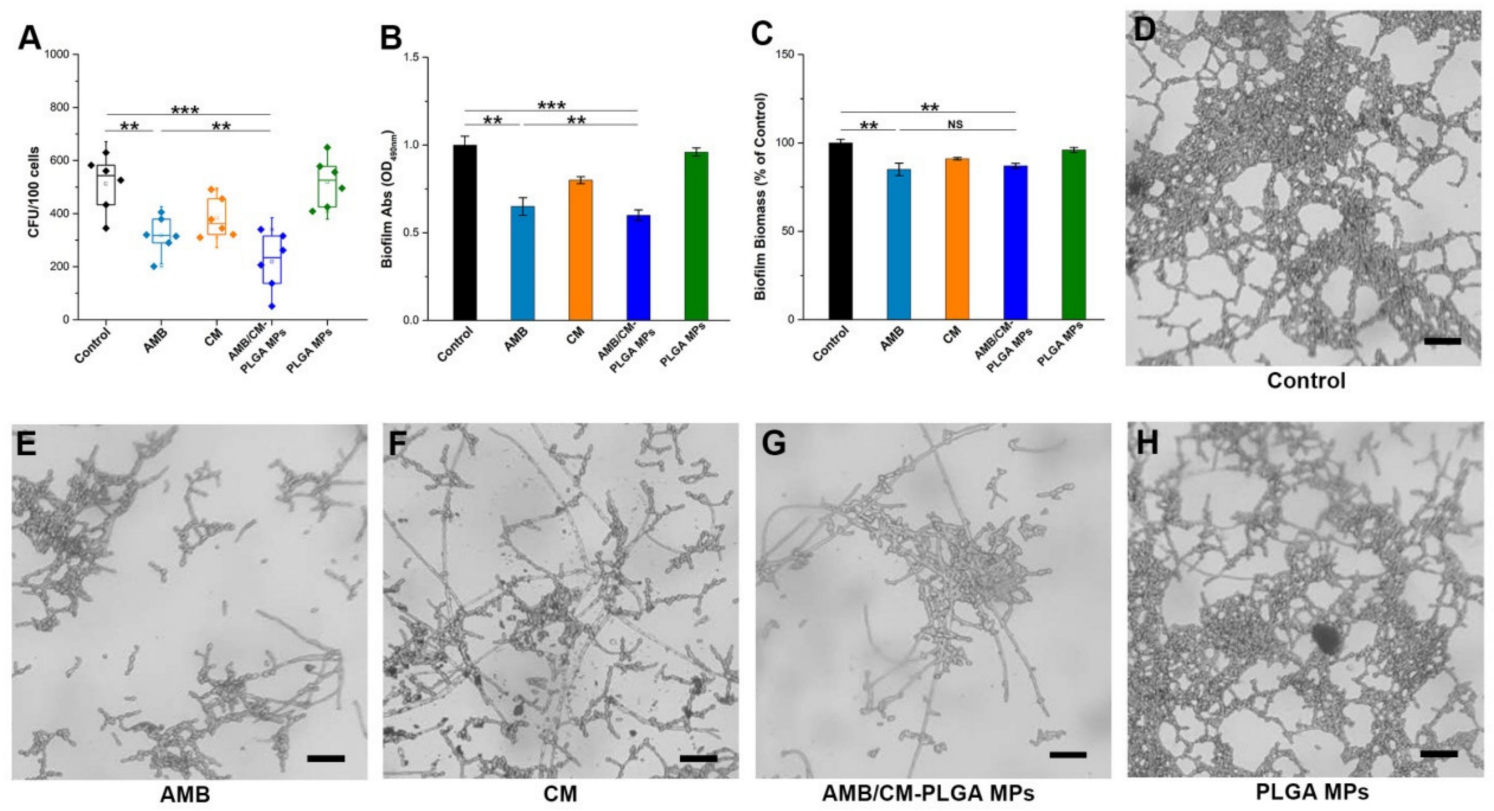
(**A**) Adhesion was inhibited significantly following treatment with AMB, CM, and AMB/CM-PLGA MPs, especially AMB/CM-PLGA MPs. (**B**) Activity of biofilm decreased significantly following treatment with AMB, CM, and AMB/CM-PLGA MPs, especially AMB/CM-PLGA MPs. (**C**) Biofilm biomass decreased significantly following treatment with AMB, CM, and AMB/CM-PLGA MPs, but there was almost no difference between AMB and the AMB/CM-PLGA MPs group. (**D**–**H**) Morphological observation of *C. albicans* at 24 h (bar = 20 μm). The final concentration of AMB employed in the AMB group and the AMB/CM-PLGA MPs group was 0.5 μg/mL. ** *p* < 0.05. *** *p* < 0.01. NS means no significance.

The tested drugs, AMB, CM, and AMB/CM-PLGA MPs, were able to inhibit the adhesion of strains to PECs. The amount of fungi in the AMB group was approximately 302 CFU/100 cells, while the amount of fungi in the AMB/CM-PLGA MPs group was approximately 215 CFU/100 cells, and they were less than the control group (525 CFU/100 cells). It can be seen that the CFU of the AMB/CM-PLGA MPs group was lower than that of the AMB group (Figure 6A).

**Scheme 1:**
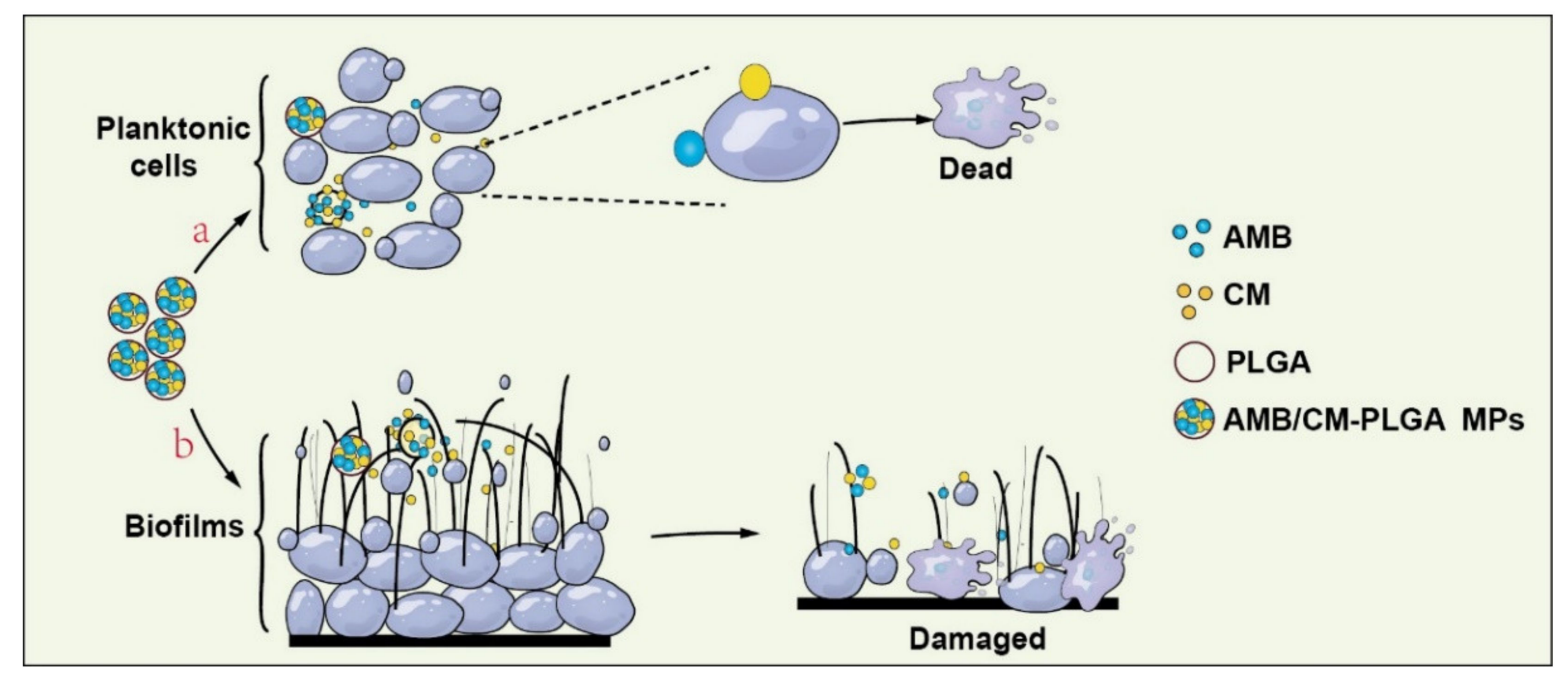
Amphotericin B (AMB) and curcumin (CM) co-loaded porous PLGA microparticles as a sustained release system for fungal planktonic cells (**a**) and biofilms (**b**).

To investigate the antifungal effect of AMB/CM-PLGA MPs on *C. albicans* ATCC 90028 biofilms, the activity of biofilms was measured by the XTT reduction method and the biomass of biofilm was measured after different treatments [23,24]. The color change displayed by the XTT reduction method was related to the biofilm activity, which was analyzed by the optical density at 490 nm. Compared to the control group, the activity of biofilms was reduced after AMB, CM, or AMB/CM-PLGA MP treatments. The inhibition rates for the AMB group, CM group, and AMB/CM-PLGA MPs group were 58.65, 76.35, and 55.64%, respectively. The activity of biofilms in the AMB/CM-PLGA MP treatment group was significantly lower than that of the AMB treatment group (Figure 6B). In addition, the total biofilm biomass of the AMB, CM, and AMB/CM-PLGA MPs treated was also significantly decreased compared with the control group, but there was almost no difference between AMB and the AMB/CM-PLGA MPs group (Figure 6C). Figure 6D–H show that the AMB/CM-PLGA MPs group had more significant inhibition of biofilm formation than other groups at 24 h when the tested agents were added at adherence. In summary, AMB/CM-PLGA MPs had a more obvious effect on adhesion and biofilm formation compared with AMB. The study procedures that the AMB/CM-PLGA MPs used as a sustained-release system to treat planktonic cells and biofilms are illustrated in Scheme 1.

### 2.7. Antimicrobial Activity In Vivo

**Figure 7:**
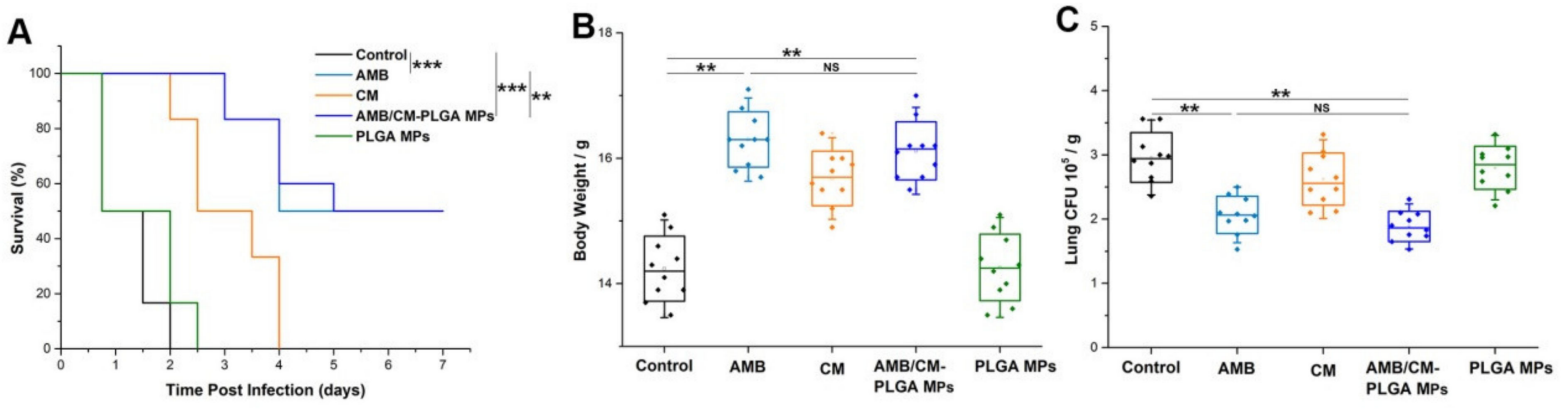
In vivo antifungal activity test. (**A**) Survival curves were monitored in the following 7 days upon *C. albicans* infection. (**B**) Body weight was measured 24 h post-infection. (**C**) Lung tissue fungal burdens were tested using CFU assay. The final concentration of AMB employed in the AMB group and the AMB/CM-PLGA MPs group was 0.5 μg/mL; the final concentrations of CM and PLGA MPs were 1.2 and 1.7 μg/mL, respectively, which were the same as those contained in the AMB/CM-PLGA MPs group. ** *p* < 0.05. *** *p* < 0.01. NS means no significance.

To further evaluate the antifungal activity of the AMB/CM-PLGA MPs in vivo, after aerosol inhalation with AMB, CM, AMB/CM-PLGA MPs or PLGA MPs, C57BL/6 mice were infected with *C. albicans* by intranasal instillation. The mortality of the mice was monitored over 7 days (Figure 7A). AMB, CM, and AMB/CM-PLGA MPs decreased animal mortality from fungal infection, especially AMB and AMB/CM-PLGA MPs. In addition, the survival rate of the AMB/CM-PLGA MPs group was slightly higher than that of the AMB group. All mice in the untreated group (control group) and PLGA MPs group died within 2.5 days after infection; CM-treated mice died at 4 days, while 50% of AMB- and AMB/CM-PLGA MPs-treated mice still survived within 7 days. Although AMB and AMB/CM-PLGA MPs protected mice from severe weight loss compared with the control group, there was no difference between them (Figure 7B). Additionally, the mice were killed when they had become moribund, the organs were removed and homogenized, and the fungal burden was determined by serial dilution on PDA plates. Both the AMB and AMB/CM-PLGA MPs groups showed significantly decreased CFU compared with the control group, but there was also no difference between them (Figure 7C), which was consistent with body weight results [13]. In vivo experiments, before and after treatment with AMB and AMB/CM-PLGA MPs, there was no significant difference in the body weight and fungal burden of lung organs, but there was a difference in the survival rate of mice. The reason may be that CM improved the immunity of mice, but the specific mechanism needs to be further verified.

AMB has a high affinity for fungal cell membrane sterols, especially ergosterol; this effect is responsible for its severe nephrotoxicity due to its interaction with the cholesterol-rich membrane of kidney cells. This toxic side effect seriously affects the clinical application of AMB. Nanotechnology has yielded promising results in developing DDS for the treatment of various diseases. These nanoscale carrier systems offer several advantages over traditional delivery systems, such as the protection of encapsulated drugs from degradation and metabolism, increased residence time, and enhanced targeting of specific cells or organs. Consistent with this, nanoparticle-based AMB formulations significantly reduced side effects and improved their therapeutic index, for example, polymer nanoparticles [25], lipid-based nanoparticles [26], and metal-based nanoparticles [27].

Compared with nanoscale carrier systems, porous microparticles show advantages in pulmonary administration. For example, porous microparticles have the characteristics of large geometric diameter and low bulk density, which can reduce the phagocytosis of alveoli and the trend of particle aggregation so as to improve atomization efficiency. At present, porous microparticles are mostly used to treat lung cancer [28]. There is no report of using porous microparticles to carry AMB. The co-loading of AMB and CM with porous microparticles was reported for the first time in this study. In summary, a porous microparticle DDS co-loaded with AMB and CM was prepared by the traditional method in this study, which is the AMB/CM-PLGA MPs. The aim is to use this DDS to reduce the toxic and side effects of AMB and better treat Candida infections, especially in the treatment of lung infections by aerosol inhalation. The particle size of AMB/CM-PLGA MPs is 47.5 ± 4.2 μm, and the particle size distribution is uniform (SPAN value = 0.79), which is suitable for the aerosol administration route. AMB/CM-PLGA MPs also have a good sustained-release effect, which can ensure a high level of drug concentration at the action site. Furthermore, during the preparation process, there were no additional chemical additives such as penetrants, foaming agents, and porogens; by optimizing ultrasonic frequency and homogenization speed, the purpose of porousness can be achieved. The step of removing chemical additives can be omitted, and the safety effect can be achieved. The porous microparticle DDS may be a novel and promising drug candidate for the treatment of pulmonary fungal infections.

## 3. Materials and Methods

### 3.1. Chemicals and Reagents and Strains

AMB, CM, and PLGA (lactide:glycolide = 50:50, Mw: 24,000~38,000 Da) were purchased from Sigma-Aldrich (St Louis, MO, USA). *C. albicans* ATCC 90028 and *C. albicans* ATCC 10231 were standard strains (ATCC: American Type Culture Collection).

### 3.2. Preparation and Characterization of AMB/CM-PLGA MPs

AMB and CM co-loaded into porous PLGA microparticles (AMB/CM-PLGA MPs) were prepared by double emulsion solvent evaporation technology [14]; 140 mg of PLGA was added to 5 mL of dichloromethane solution and then sonicated for 30 s at an amplitude of 50%; 20 mg AMB and 40 mg CM were dissolved in 2 mL of acetone solution; then, this solution was added to the above dichloromethane solution, and 1% PVA solution was added while sonicating. Then, the above emulsion obtained was added to 1% PVA solution with high-speed stirring. Afterward, mechanical stirring at 300 rpm was continued for 4 h to remove the solvent. The solidified MPs were centrifuged (10,000 rpm) and washed three times with DI to remove PVA and non-incorporated drugs. Finally, the washed MPs were lyophilized and stored in a vacuum for further analysis. The surface morphology of AMB/CM-PLGA MPs was characterized by scanning electron microscopy (SEM) (JEOL, Tokyo, Japan). To characterize the homogeneity of the prepared microparticles, SPAN values were investigated using the following formula:

SPAN = (D90 − D10)/D50.

D10, D50, and D90 were the particle sizes at 10%, 50%, and 90% of the sample volume, respectively.

### 3.3. Drug Loading Content (DLC) and Drug Loading Efficiency (DLE)

The DLC and DLE of AMB and CM were measured by UV–vis spectrophotometer at 420 and 410 nm, respectively. Take CM as an example; a calibration curve was established using standard solutions of CM. The calibration curve was linear between 500 ng and 3.0 μg with good linearity (R2 = 0.9988). Lyophilized AMB/CM-PLGA MPs (2 mg) were dissolved in 1 mL of methanol completely to extract CM to methanol for the DLC and DLE. The samples in methanol were gently shaken on a shaker for 24 h at 37 °C to leach out CM entirely. Then the solutions were centrifuged at 10,000 rpm for 20 min, and the supernatant was gathered. The supernatant (100 μL) was diluted to 2 mL for DLC and DLE detection using a UV–vis spectrophotometer at 410 nm. The value was calculated using the following formulas:

DLC (wt%) = (weight of loaded CM/weight of AMB/CM-PLGA MPs)%

DLE (wt%) = (weight of loaded CM/weight of feeding CM)%

The DLC and DLE of AMB in AMB/CM-PLGA MPs were determined by UV absorption at 420 nm, and the measurement methods are similar to CM.

### 3.4. Determination of Drug Release

The drug release of AMB or CM from AMB/CM-PLGA MPs was determined using a modified dialysis bag method [17,29]. Briefly, AMB/CM-PLGA MPs were placed in a pre-treated dialysis bag and immersed in PBS containing 15% (*v*/*v*) ethanol while being shaken at 37 °C and 60 rpm. At a preset time point (1, 2, 3, 4, 5, 6, 12, 18, 24, 36, 48, 60, 72, 96, 120, 144, and 168 h), the released drug was harvested and replaced with the same volume of PBS. The content of released AMB/CM-PLGA MPs was detected by a UV spectrometer at 420 nm for AMB or at 410 nm for CM. All assays were carried out three times in triplicate.

### 3.5. Hemolysis Determination

The study of hemolytic activities was investigated using rabbit red blood cells (rRBCs) assay, as previously described [17]. Briefly, fresh rabbit blood obtained from the Experimental Animal Center of Guangzhou Medical University was diluted using physiological saline. rRBCs were harvested by centrifugation and washed carefully. Then, the suspension of rRBCs was added into AMB or AMB/CM-PLGA MPs solution and blended gently. The samples were collected and added to 96-well plates. The content of free hemoglobin was detected at 576 nm using a microplate reader at 1, 2, 4, and 6 h, respectively. The group treated with 1% Triton-X was used as a positive control, and the group treated with PBS was used as a negative control. The value was calculated using the following formulas:

Hemolysis (%) = [(OD576 nm of treated sample − OD576 nm of negative control)/(OD576 nm of positive sample − OD576 nm of negative control)] × 100%.

### 3.6. Nephrotoxicity Determination

The study of nephrotoxicity was determined in a mouse model, as previously described [30]. Healthy BALB/c mice were randomly divided into 5 groups of 4 mice each. AMB or AMB/CM-PLGA MPs was given at a dose of 0.26 or 0.8 mg/kg for three consecutive days. The blood sample was collected from the mice to measure levels of blood creatinine (CRE) and blood urea nitrogen (BUN).

### 3.7. Minimum Inhibitory Concentration (MIC) Assay

The MIC assay was determined for the tested fungi based on clinical and laboratory standards institute guidelines (M38-A) [13]. Considering that both AMB and CM were hydrophobic drugs, DMSO was used to dissolve these compounds, and then PBS (pH 7.4) was used to dilute them to the concentration required for the test while ensuring that the final concentration of DMSO was not higher than 0.1%. For the configuration of the AMB/CM-PLGA MPs solution of the test sample, PBS (pH 7.4 and 0.5% Tween 20) was used to prepare the emulsion. Initial concentrations of CM and AMB/CM-PLGA MPs were 512 μg/mL, and AMB was 16 μg/mL. The final concentration of the tested strains was prepared at 1.0~2.0 × 10^4^ CFU/mL and performed in 96-well plates at 37 °C for 24 h. The MIC was defined as the lowest reagent concentration that inhibited the growth of the tested strain. All assays were carried out three times in triplicate.

### 3.8. Growth Kinetics of C. albicans

The study on the kinetics of fungal growth was investigated for *C. albicans*, as previously described [31]. Briefly, the tested strains were inoculated into potato dextrose broth (PDB) containing MIC/2 concentrations of tested agents at 37 °C for 30 h. The optical density values were measured by a UV spectrometer at 560 nm, and the growth curves were drawn based on the measured values. All assays were carried out three times in triplicate.

### 3.9. Inhibition of Adhesion Assay

The inhibition of the adhesion assay was conducted as described previously [27]. Briefly, *C. albicans* were treated by AMB, CM, and AMB/CM-PLGA MPs at their MIC value for 1 h at 37 °C and were then incubated with MLE-12 cells for another 1 h at 37 °C. The number of *C. albicans* that adhered to cells was quantified by colony formation units assay from 100 randomly collected and lysed cells.

### 3.10. XTT/Menadione Reduction Assay

A semi-quantitative measurement of fungal biofilm formation was obtained from the XTT/menadione reduction assay [23]. Briefly, 100 μL tested strains suspension (1 × 10^6^ CFU/mL) was added into selected wells and incubated at 37 °C; 100 μL tested agents were added into wells with biofilm and incubated at 37 °C for 24 h. Then, 100 μL of XTT/menadione solution was added into wells where the biofilm was formed and incubated at 37 °C. Finally, the supernatants were transferred to a new plate and the optical density was measured at 490 nm. All assays were carried out three times in triplicate.

### 3.11. Dry Mass Measurement of C. albicans Biofilms

The total biomass of *C. albicans* biofilms was determined, as previously described [32]. Briefly, *C. albicans* were inoculated into PDB to increase the number of fungi. Fungal cells were collected and then treated with AMB, CM, and AMB/CM-PLGA MPs at their MIC value. The above mixture was added to a 24-well plate containing a disc with a diameter of 14 mm and then incubated at 37 °C. The liquid was removed from the plate, and then the discs were incubated in PDB for 72 h. After treatment, the discs were dried to a constant weight at 80 °C. The dry weight of the biofilm was calculated. All assays were carried out three times in triplicate.

### 3.12. Morphological Observation of C. albicans Biofilms

In order to analyze the influence of drugs on the structure of biofilms, morphological observation of *C. albicans* biofilms was performed using a Motic BA410E microscope (Motic, Xiamen, China). The operation of biofilm formation was the same as above. Briefly, fungal cells were harvested and then treated with AMB, CM, or AMB/CM-PLGA MPs at their MICs for 1 h. The liquid was removed from the plate, and then the discs were incubated in PDB for 72 h. The discs were washed with PBS to remove planktonic cells and observed by microscope.

### 3.13. In Vivo Antifungal Activities

C57BL/6 mice (8–12 weeks old) with an average body weight of 14 ± 2 g were purchased from Guangdong Experimental Animal Center (Guangzhou, China). All mice were maintained in plastic cages, with free access to food and water, and housed at 22–25 °C. All mice were randomly assigned to the experimental group or control group and raised together, and the experiment was not carried out blindly. AMB, CM, AMB/CM-PLGA MPs, and PLGA MPs were inhaled by the experimental group or control group, respectively, and it was ensured that the final concentration of AMB employed in the AMB group and the AMB/CM-PLGA MPs group was 0.5 μg/mL, the final concentrations of CM and PLGA MPs were 1.2 and 1.7 μg/mL, respectively, which were the same as those contained in the AMB/CM-PLGA MPs group. For pulmonary infections, 1 × 10^6^ CFU of *C. albicans* were administered to the nasal cavity of isoflurane-anesthetized mice. The mice were killed when they had become moribund, the organs were removed and homogenized, and the fungal burden was determined by serial dilution on potato dextrose agar (PDA) plates. All animal experiments were performed in accordance with the Guide and Care and Use of Laboratory Animals from the National Institutes of Health (NIH) and ARRIVE and approved by the Experimental Animal Ethics Committee of Guangzhou Medical University (Audit Form No.: 2019-207).

### 3.14. Statistical Analysis

One-way ANOVA (Tukey’s post hoc) or Student’s t-test was used for statistical analysis. The symbols “**” and “***” denote *p*-values less than 0.05 and 0.01, respectively. *p* < 0.05 was considered statistically significant.

## 4. Conclusions

In this work, AMB/CM-PLGA MPs were successfully prepared by the traditional double emulsion method. The drug delivery system reduced the toxic side effects of AMB while improving the activity against *C. albicans* in inhibiting adhesion and biofilm formation, which may be a novel and promising drug candidate for the treatment of fungal infections.

## Figures and Tables

**Table 1:** Investigation of the drug loading content (DLC) and drug loading efficiency (DLE) of AMB/CM-PLGA MPs.

	Feeding Ratio (PLGA:AMB:CM)	AMB			CM		
		Feeding AMB Content (wt%)	DLC (wt%)	DLE (%)	Feeding CM Content (wt%)	DLC (wt%)	DLE (%)
A	18:1:1	2	2.25	45.0	2	3.05	61.0
B	16:1:3	2	2.41	48.2	6	10.3	68.7
C	14:2:4	4	6.08	60.8	8	14.5	72.5
D	12:2:6	4	6.12	61.2	12	19.2	64.0

**Table 2:** Minimum inhibitory concentrations (MICs) of AMB, CM, and AMB/CM-PLGA MPs against *C. albicans* ^a^.

Tested Strains	MIC (μg/mL)		
	AMB	CM	AMB/CM-PLGA MPs
*C. albicans* ATCC 90028 ^b^	0.5	128	8 (0.4864) ^c^
*C. albicans* ATCC 10231 ^b^	0.5	256	8 (0.4864) ^c^

Notes: ^a^ Each value is the mean of three independent experiments carried out in triplicate. ^b^ American type culture collection (USA) strains. ^c^ This value (8 μg/mL) was the content of AMB/CM-PLGA MPs. Since the DLC of AMB in AMB/CM-PLGA MPs was 6.08%, the value (8 μg/mL) actually contained AMB at 0.4864 μg/mL.

## Data Availability

Not applicable.
